# Budgetary Impact of Treating Acute Promyelocytic Leukemia Patients with First-Line Arsenic Trioxide and Retinoic Acid from an Italian Payer Perspective

**DOI:** 10.1371/journal.pone.0134587

**Published:** 2015-08-12

**Authors:** Morgan Kruse, Rebecca Wildner, Gisoo Barnes, Monique Martin, Udo Mueller, Francesco Lo-Coco, Ashutosh Pathak

**Affiliations:** 1 Optum, Cambridge, Massachusetts, United States of America; 2 Teva Pharmaceuticals, Frazer, Pennsylvania, United States of America; 3 MapiGroup, Uxbridge, United Kingdom; 4 Teva Pharmaceuticals, Ulm, Germany; 5 Department of Biomedicine and Prevention, University Tor Vergata, Rome, Italy; Kingston University London, UNITED KINGDOM

## Abstract

The objective of this study was to estimate the net cost of arsenic trioxide (ATO) added to all-*trans* retinoic acid (ATRA) compared to ATRA plus chemotherapy when used in first-line acute promyelocytic leukemia (APL) treatment for low to intermediate risk patients from the perspective of the overall Italian healthcare systemA Markov model was developed with 3 health states: stable disease, disease event and death. Each month, patients could move from stable to disease event or die from either state. After a disease event, patients discontinued initial treatment and switched to the other regimen as second-line therapy. Treatment regimens, efficacy and adverse events were derived from published sources and expert opinion; unit costs were collected from standard Italian sources. Clinical outcomes and costs for pre-ATO and post-ATO scenarios were combined with population and product utilization information to calculate the total budgetary impact using a 3-year time horizon; one-way sensitivity analyses were conducted. Three-year cumulative pharmacy costs for ATO+ATRA were €46,700 per-patient versus €6,500 for ATRA+chemotherapy; however, medical costs for ATO+ATRA were €12,300 per-patient versus €30,200 for ATRA+chemotherapy. The total budgetary impact was estimated to be an additional €127,300, €312,500 and €477,800 in the first, second and third years, respectively. The model was most sensitive to changes in the cost of the ATO+ATRA regimen during the consolidation phase. Budgetary impact models are valuable to payers making formulary decisions regarding the access and affordability of new medicines. The cost of treatment analysis showed that pharmacy costs for ATO+ATRA were higher than for ATRA+chemotherapy, while all other evaluated costs were lower for ATO+ATRA treated patients. The average budgetary impact was €305,900 per year overall, representing a 3.5% increase. Further research is needed to determine the cost-effectiveness of ATO+ATRA compared to the current first-line standard of care in APL.

## Introduction

Acute promyelocytic leukemia (APL) is a distinct subtype of acute myeloid leukemia (AML), defined by a balanced chromosomal translocation t(15,17) resulting in the fusion of the promyelocytic leukemia (*PML*) and retinoic acid receptor-α (*RAR-α*) genes resulting in an aberrant oncogenic protein which blocks myeloid differentiation at the promyelocyte state. It is estimated that APL accounts for between 5 and 12% of all AML cases, depending on the country or region [1]. The median age of diagnosis for APL is 40 years compared to 65 years for AML with approximately 80% of people with APL aged between 15 and 55 years [1].

APL was once the most fatal of the acute leukemias, but treatment was revolutionized in the 1980s by the introduction of all-*trans* retinoic acid (ATRA) which, given in association with chemotherapy transformed APL into the most highly curable acute leukemia [2]. Previous multicenter trials examining the efficacy of ATRA used in combination with chemotherapy have reported remission rates as high as 95% and long-term disease-free survival as well as cure rates exceeding 80% [2–7]. The combination of ATRA and chemotherapy for induction (initial therapy usually lasting about 1 month) followed by consolidation (1 to 4 monthly cycles of additional treatment) is currently considered the standard of care [2,8]. Specifically, ATRA plus anthracycline-based chemotherapy for induction and consolidation followed by maintenance with ATRA and low-dose chemotherapy is currently the standard of care for first-line therapy in Italy [7].

In 2002, arsenic trioxide (ATO; Trisenox, manufactured by Teva Pharmaceuticals) was approved in the EU (US approval first occurred in 2000) for the treatment of patients with APL who are refractory to, or have relapsed from, previous treatment with ATRA and anthracycline chemotherapy. Early studies reported that approximately 85% of relapsed patients initially treated with ATRA achieved sustained molecular remission with second-line ATO therapy [9–11]. In fact, studies have reported complete remission rates of approximately 80–90% and overall survival (OS) rates of 50–70% at 1 to 3 years [11–14].

Studies have also reported that patients with newly diagnosed APL benefit from ATO therapy either alone or in combination with ATRA [15–17]. Specifically, clinical trials have reported that patients receiving ATO plus ATRA induction therapy experienced fewer relapses, achieved complete remission faster, had lower hematologic toxicity, and significantly better 2-year OS rates compared to patients receiving standard ATRA-chemotherapy [18–20]. The use of ATO in conjunction with chemotherapy during consolidation therapy is also highly effective [21].

A budgetary impact model was developed to estimate the financial impact of including ATO on the national formulary in Italy for the treatment of first-line APL. This type of analysis is critical for payers to fully understand what the potential net impact of a new health technology will be, including expenditures on pharmacy, medical (including hospitalization and physician visits) and other related healthcare costs.

The objective of this study was to estimate the net cost of ATO added to ATRA compared to ATRA plus anthracycline-based chemotherapy when used in first-line APL treatment from the perspective of the overall Italian healthcare system which only includes public payers.

## Materials and Methods

This paper describes an interactive model that estimates the changes in drug and medical costs due to the introduction of ATO (added to ATRA) as a first-line treatment for patients with APL in Italy. It has been developed in accordance with the International Society of Pharmacoeconomics and Outcomes Research (ISPOR) Principles of Good Practice for Budget Impact Analysis [22], as well as the Guidelines for Economic Evaluations in Italy [23]. The model uses a base case set of variables based on national data, published clinical trial evidence and expert clinical opinion; no patient-level identifiable data were used. The clinical outcomes and costs for the pre-ATO and post-ATO scenarios are combined with population and product utilization information to calculate the total budgetary impact associated with the introduction of ATO.

### Model Structure

**Fig 1:**
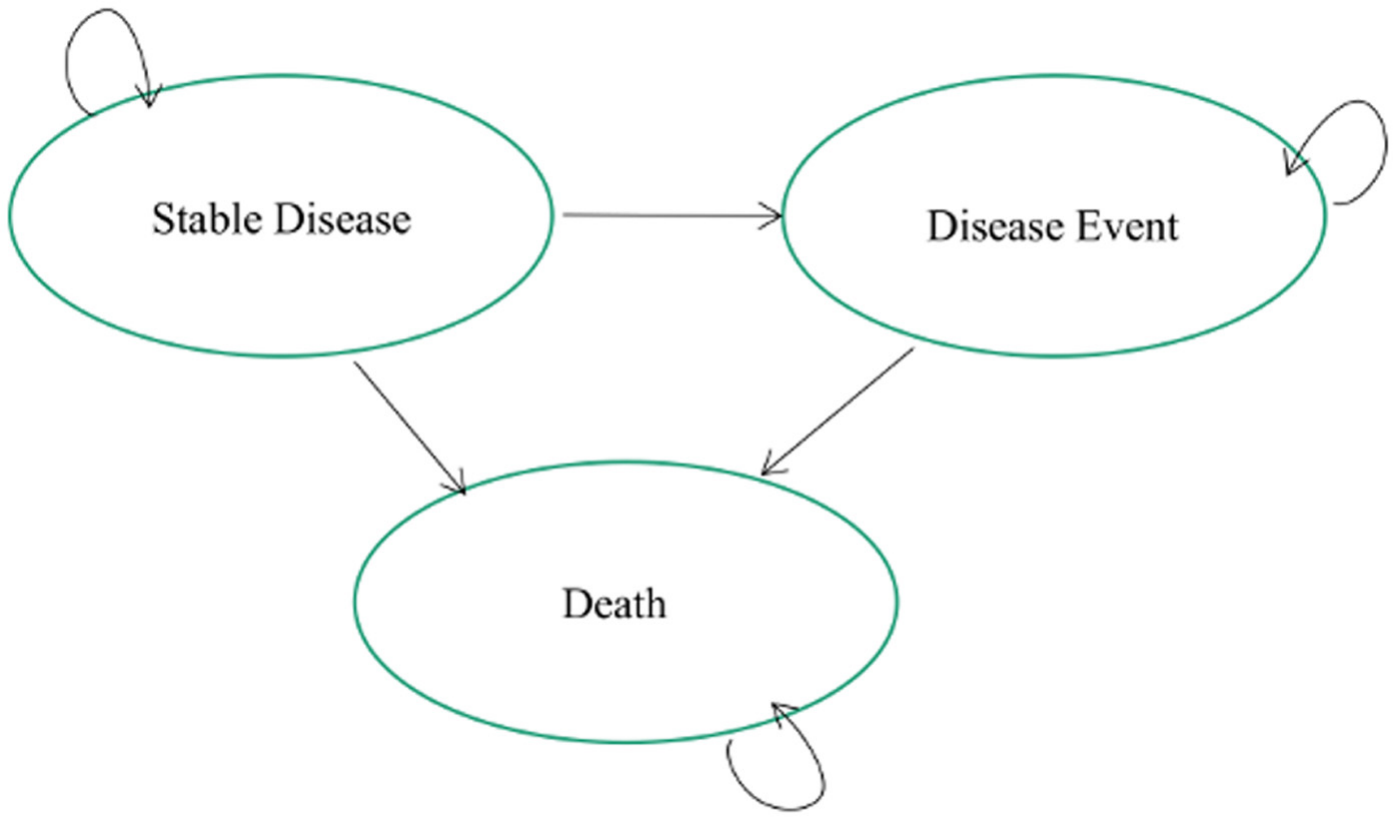
Markov model of first-line chemotherapy with three disease states.

A Markov decision analysis model was constructed in Microsoft Excel 2010 with 3 health states: stable disease, disease event and death. In a Markov model, a patient is always in one of a finite number of discrete health states with movement between the states during each cycle dictated by transition probabilities. The Markov model is used to estimate the time spent in each health state, as well as the survival and other outcomes accrued over time. An overview of the model structure is shown in Fig 1 and the data inputs can be found in S1 File. The model consists of monthly cycles that characterize the outcomes of treatment for each annual patient cohort over 3 years. A 3-year time horizon was utilized to reflect the short-term nature of the budgetary cycle. The analysis assumes that a second cohort of newly eligible patients enters the model in year 2; as such, the patient population in year 2 includes both new patients and surviving patients from year 1. Subsequently, an additional cohort enters the model in the third year and is followed for that year in addition to the surviving patients from cohorts 1 and 2. A half-year correction was employed to approximate a uniform distribution throughout the year (i.e., all new patients start the cycle on July 1st instead of January 1st). Only costs that are realized in each of the 3 years in the model time horizon are included in the analysis and were not discounted based on recommendations from the ISPOR guidelines for budget impact analysis [22]

The model consists of one-month cycles that characterize the outcomes of treatment for each annual patient cohort over three years. At any point, patients fall into one of three mutually exclusive states: stable disease, disease event, and death. All patients begin the model in stable disease, and can move to either disease event or death in each cycle, or remain in stable disease.

All patients enter the model with stable disease and each month could move from stable to disease event or die from either state due to the effects of APL or treatment-related mortality. In the model, stable patients may experience hematologic or molecular relapse and thus have a disease event. Additionally, the failure to achieve hematologic complete remission at the end of induction or molecular complete remission at the end of 3 consolidation cycles constitutes a disease event. Hematologic remission refers to leukemic cell clearance from the marrow below 5% as evaluated by morphology, i.e. microscopy. Molecular remission refers to a more profound level of leukemic cell clearance (usually below 1/10,000 normal cells as evaluated by more sophisticated techniques (e.g. molecular biology) that look at DNA, RNA or protein biomarkers of that particular leukemia.

After a disease event, patients discontinue initial treatment and switch to the other regimen as second-line therapy [24], although they remain in the disease event state. Mortality is based on the transition probability for patients who have had a disease event (as second-line patients are first-line progressed patients). Third-line, or salvage, treatment is not modelled. In addition to the state transitions detailed above, patients may experience adverse events (AEs) related to treatment with either regimen (neutropenia, thrombocytopenia, hepatic toxicity, and fever of unknown origin were included based on clinical trial data) [19]. These were deemed to have only transitory impact on treatment, and therefore no changes aside from medical treatment costs were linked to AEs.

### Eligible Population

A budgetary impact model starts with the overall population of interest and then narrows down to the population eligible for the new therapy. The present model included adult patients (at least 18 years of age) who had been newly diagnosed with APL and were of low to intermediate risk based on white blood cell count, as treatment guidelines differ for high-risk patients [2]. Population estimates are summarized in Table 1. Beginning with the total population of Italy from the Italian National Institute of Statistics [25], the annual age-adjusted incidence of AML was taken from the Italian National Cancer Registry [26]. The proportion of patients with the APL subtype was taken from Lengfelder and colleagues [24,27] as there were no robust estimates of the APL proportion within the Italian AML population. As only low to intermediate risk patients are eligible for treatment with ATO, the percentage of patients in these risk categories was estimated from a study of European patients [28]. Finally, it was assumed that 100% of eligible patients would receive first-line treatment.

**Table 1:** Population Estimates.

Parameter	Estimated Value	Eligible Population (N)
Italian population [25]	60,021,955	60,021,955
Annual age-adjusted incidence of AML [26]	3.2 per 100,000	1921
Proportion of AML that is APL [24,27]	6.0%	115
APL patients who are low to intermediate risk [28]	71%	82
Proportion of eligible APL patients who receive first-line treatment†	100%	82

^†^Assumption.

### Clinical Parameters and Survival Estimates

The main clinical parameters included in the model were event-free survival (EFS) and OS. Survival estimates were obtained from a phase 3 multicenter clinical trial that compared ATO+ATRA versus a standard ATRA+chemotherapy regimen [19]. OS and EFS were reported in Kaplan-Meier curves over the course of approximately 54 months. The primary study outcome was EFS at 2 years after diagnosis, with an event defined as: 1) no achievement of hematologic complete remission after induction therapy, 2) no achievement of molecular complete remission after 3 consolidation courses, 3) molecular relapse, 4) hematologic relapse, or 5) death. The same definition was used to determine a disease event / death in the model.

The monthly estimates from the Kaplan-Meier curves for EFS and OS were used to estimate the following 3 transition probabilities: from stable disease to disease event, from stable disease to death, and from disease event to death (see Table 2). Calibration was conducted using Microsoft Excel Solver where the calibration process simultaneously manipulated the transition probabilities such that the deviation between the observed clinical trial data and the predicted data (model-produced outcomes) was minimized. The calibration process has 2 steps:

- Step 1: for each set of Kaplan-Meier curves, a mathematically based graphical curve-fitting method using Microsoft Visual Basic for Applications in Excel was used to estimate the proportion of patients who were alive (from OS) and patients who were alive without a disease event (EFS) at monthly time intervals.
- Step 2: in the Markov portion of the model, for each model comparator, the proportion of patients who were alive (from OS) and patients who were alive without a disease event (EFS) during each cycle was tabulated.

**Table 2:** Clinical Transition Probabilities.

Parameter	Transition Probability
**ATO+ATRA Regimen** [19]	
Probability of a disease event from stable disease	0.00084
Probability of death from a disease event	0.00058
Probability of disease death from stable disease (including remission)	0.00058
**ATRA+Chemotherapy Regimen** [19]	
Probability of a disease event from stable disease	0.00202
Probability of death from a disease event	0.00444
Probability of disease death from stable disease (including remission)	0.00444

**Fig 2:**
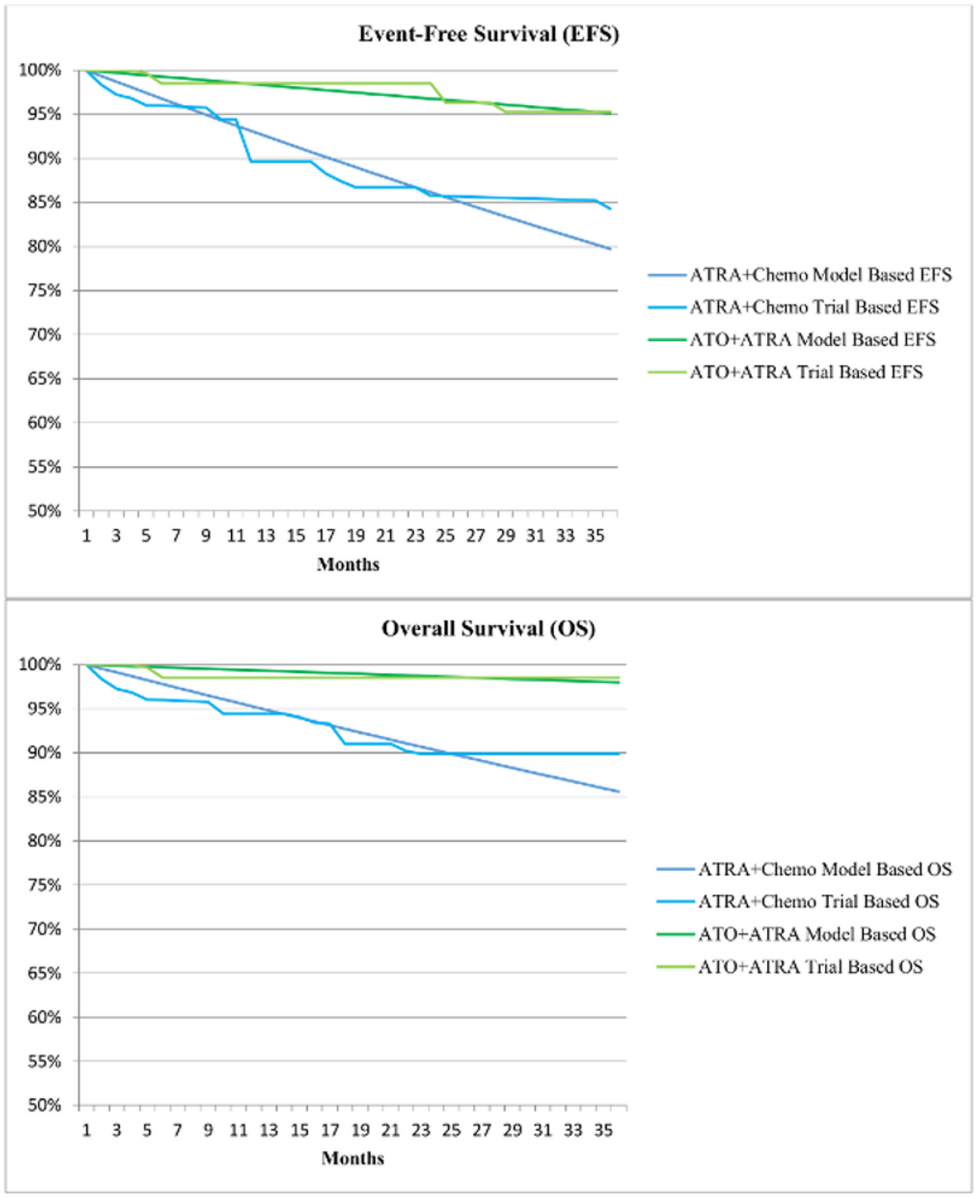
Model calculated survival curves overlaid with observed clinical trial data [19]. The survival curves derived from the estimated model were similar to those found in previous reported clinical trial data [19]

Microsoft Excel Solver calculated the transition probabilities such that the sum of the percent absolute value difference between EFS and OS in step 1 and step 2 was as small as possible. The model’s calculated survival curves lined up well with the data reported in the clinical trial (see Fig 2).

The probabilities of AEs in each treatment phase for both regimens were also taken from Lo-Coco and colleagues [19]. Data were collected on hematologic toxicity (neutropenia, thrombocytopenia, and fever of unknown origin), as well as non-hematologic toxicity (including hepatic toxicity, QTc prolongation, gastrointestinal toxicity, and oral toxicity). For the purposes of this paper, only AEs that required extensive treatment and thus incurred substantial cost were included (see Table 3).

**Table 3:** Adverse Event Probabilities by Treatment Phase.

Parameter		Probability of Grade 3 or 4 Adverse Event	
Treatment Phase	Adverse Event	ATO+ATRA Regimen [19]	ATRA+Chemotherapy Regimen [19]
Induction	Neutropenia	46.0%	79.0%
	Thrombocytopenia	59.0%	88.0%
	Hepatic toxicity	2.5%	0.4%
	Fever of unknown origin	1.1%	9.8%
Consolidation	Neutropenia	6.0%	35.0%
	Thrombocytopenia	6.0%	18.0%
	Hepatic toxicity	2.5%	0.4%
	Fever of unknown origin	1.1%	9.8%
Consolidation second cycle	Neutropenia	6.0%	76.0%
	Thrombocytopenia	6.0%	65.0%
	Hepatic toxicity	2.5%	0.4%
	Fever of unknown origin	1.1%	9.8%
Consolidation third cycle	Neutropenia	4.0%	25.0%
	Thrombocytopenia	3.0%	15.0%
	Hepatic toxicity	2.5%	0.4%
	Fever of unknown origin	1.1%	9.8%

### Product Utilization

Marketplace dynamics are important to budgetary impact models in terms of existing product utilization and the effect of the new therapy on this utilization. The rate of the new therapy’s adoption following introduction is also key. The budgetary impact of including ATO on the Italian national formulary is estimated by comparing the pre-ATO reference scenario with the post-ATO new scenario. The only first-line regimen currently approved for treating APL in Italy is ATRA plus anthracycline-based chemotherapy; therefore, this regimen accounts for 100% of the market share in the pre-ATO reference scenario. Expert clinical opinion was consulted to determine the rate at which patients would switch to the ATO+ATRA regimen if it was approved and reimbursed in the new scenario. Assumptions were based on previous experience with the GIMEMA group as there are no other multicenter groups in Italy that deal with treatment of APL and virtually all Italian patients with APL who do not experience early death and are healthy would have entered a GIMENA trial. The study by Lo-Coco and colleagues [19] has the potential to be practice-changing and practice may be changed in Italy more than elsewhere because data were generated by Italian hematologists. In the first year, the assumption is that 10% of patients would be treated with ATO+ATRA, followed by 20% in the second year and 30% in the third year.

### Costs

Cost estimates were collected with the input of an Italian clinical expert, who provided guidance as to the appropriate resources in regular clinical practice treating patients with APL (see Table 4). The unit costs for procedures and tests were taken from the Italian Ministry of Health’s 2013 Tariffs [29]. Estimates for chemotherapy-related pharmacy acquisition costs were obtained from the 2014 reimbursement lists and class A and H drug lists of the Italian National Drug Agency (AIFA) [30–32]; Teva Pharmaceuticals supplied the price for ATO [33]. Medical costs attributed to patients in each treatment phase differed by regimen: ATRA combined with chemotherapy included induction, consolidation, and maintenance treatment phases, whereas ATO plus ATRA included only induction and consolidation phases. Although the total second-line regimen costs are included in Table 4, only costs that occur prior to the end of the model’s 3-year time horizon are included for patients on second-line treatment (e.g., if a patient starts second-line treatment in year 3 of the model, only the year 3 costs are included even if treatment continues for over a year).

**Table 4:** Pharmacy, Medical and Adverse Event Costs (Model Inputs).

Parameter	Cost	Timing of Cost
**ATO+ATRA Regimen**		
First-line pharmacy 32, [33]	€46,814	Per-cycle, amount here is the total
Induction phase: stable disease (medical) [29]	€7,585	Per-cycle (1 induction cycle)
Consolidation phase: stable disease (medical) [29]	€398	Per-cycle
Maintenance phase: stable disease (medical) [29]	N/A	Per-cycle
Post-treatment phase: stable disease (medical) [29]	€94	Per-cycle
Disease event (medical) [29]	€430	One-time
Second-line (pharmacy and medical combined)^†^ [29], 31	€57,185	Per-cycle
**ATRA+Chemotherapy Regimen**		
First-line pharmacy 30	€6,832	Per-cycle, amount here is the total
Induction phase: stable disease (medical) [29]	€8,098	Per-cycle (1 induction cycle)
Consolidation phase: stable disease (medical) [29]	€6,128	Per-cycle
Maintenance phase: stable disease (medical) [29]	€173	Per-cycle
Post-treatment phase: stable disease (medical) [29]	€94	Per-cycle
Disease event (medical) [29]	€430	One-time
Second-line (pharmacy and medical combined)^†^ [29], 31	€37,472	Per-cycle
**Adverse Event (Treatment Phase)**		
Neutropenia (induction) [29]	€206	Per-cycle (1 induction cycle)
Thrombocytopenia (induction) [29]	€331	Per-cycle (1 induction cycle)
Hepatic toxicity (induction) [29]	€222	Per-cycle (1 induction cycle)
Fever of unknown origin (induction) [29]	€202	Per-cycle (1 induction cycle)
Neutropenia (consolidation) [29]	€66	Per-cycle
Thrombocytopenia (consolidation) [29]	€62	Per-cycle
Hepatic toxicity (consolidation) [29]	€61	Per-cycle
Fever of unknown origin (consolidation) [29]	€62	Per-cycle

^†^The total cost (pharmacy and medical combined) differs by the cycle of disease event in the model; the sum displayed here is the total for a patient in the first treatment cycle.

### Model Outcomes

The budgetary impact analysis estimates the costs associated with changing treatment patterns, such as differential survival and toxicity rates, resulting from the introduction of ATO (added to ATRA) as a first-line APL therapy. The per-patient total and component costs associated with treating APL with a first-line ATO+ATRA regimen are reported and compared to treatment with a first-line ATRA+chemotherapy regimen. The total budgetary impact is then presented for the entire Italian patient population, by year.

### Sensitivity Analyses

A series of one-way (deterministic) sensitivity analyses were conducted to test the impact of changing specific input parameter values or model assumptions on the results. Scenarios with the largest (positive or negative) impact on the third-year overall net costs are reported in a tornado diagram. These alternative scenarios varied the base case input by +/-25% for over 60 variables, including clinical and cost parameters.

## Results

The annual and cumulative per-patient treatment costs starting in year one for each regimen are presented in Table 5. The cumulative 3-year pharmacy costs for the ATO+ATRA regimen were estimated to be €46,700 per-patient versus €6,500 for the ATRA+chemotherapy regimen. In contrast, the cumulative 3-year non-pharmacy (i.e., medical, AE and disease event) costs associated with the ATRA+chemotherapy regimen were €32,300 per-patient compared with €13,300 for the ATO+ATRA regimen; the direct medical component accounted for the majority of these costs (€30,200 for ATRA+chemotherapy versus €12,300 per-patient for ATO+ATRA).

**Table 5:** Annual and Cumulative Per-Patient Treatment Costs (Model Estimated Outputs).

Cost Component	Year 1	Year 2	Year 3	Cumulative
**ATO+ATRA Regimen**				
Pharmacy	€36,800	€9,900	€0	€46,700
Medical	€9,600	€1,700	€1,000	€12,300
Adverse event	€300	€0	€0	€300
Disease event	€100	€300	€300	€700
**Total**	€46,800	€11,900	€1,300	€60,000
**ATRA+Chemotherapy Regimen**				
Pharmacy	€3,700	€1,600	€1,200	€6,500
Medical	€26,600	€1,900	€1,700	€30,200
Adverse event	€600	€0	€0	€600
Disease event	€200	€1,300	€1,200	€1,500
**Total**	€31,100	€4,800	€4,100	€38,800

Cumulative patient counts by health state (stable disease, disease event, or death) for both the current (pre-ATO) and new (post-ATO) scenarios, starting in year one, are presented in Table 6. The cumulative numbers of patients with a disease event (n = 10) or dead (n = 22) were lower in the new (post-ATO) scenario as compared to the current (pre-ATO) scenario (n = 11 and n = 27, respectively).

**Table 6:** Cumulative Patient Counts by Health State at Year End (Model Estimated Outputs).

Total Patients†	Year 1	Year 2	Year 3
Current scenario (pre-ATO)			
Stable disease	79	151	207
Disease event	1	4	11
Dead	2	8	27
New scenario (post-ATO)			
Stable disease	79	153	213
Disease event	1	3	10
Dead	1	8	22

^†^The total number of patients may not add up to 82 per year due to rounding.

The overall yearly budgetary impact of including ATO as a component of first-line treatment for APL on the overall Italian healthcare system is presented in Table 7. The total impact was €127,300 (representing a 5% increase) in the first year, €312,500 (11% increase) in the second year and €477,800 (15% increase) in the third year.

**Table 7:** Yearly Budgetary Impact (Model Estimated Outputs).

Scenario	Year 1	Year 2	Year 3
Current scenario (pre-ATO)	€2,548,300	€2,940,200	€3,259,900
New scenario (post-ATO)	€2,675,600	€3,252,700	€3,737,700
**Budgetary Impact**	**€127,300**	**€312,500**	**€477,800**

**Fig 3:**
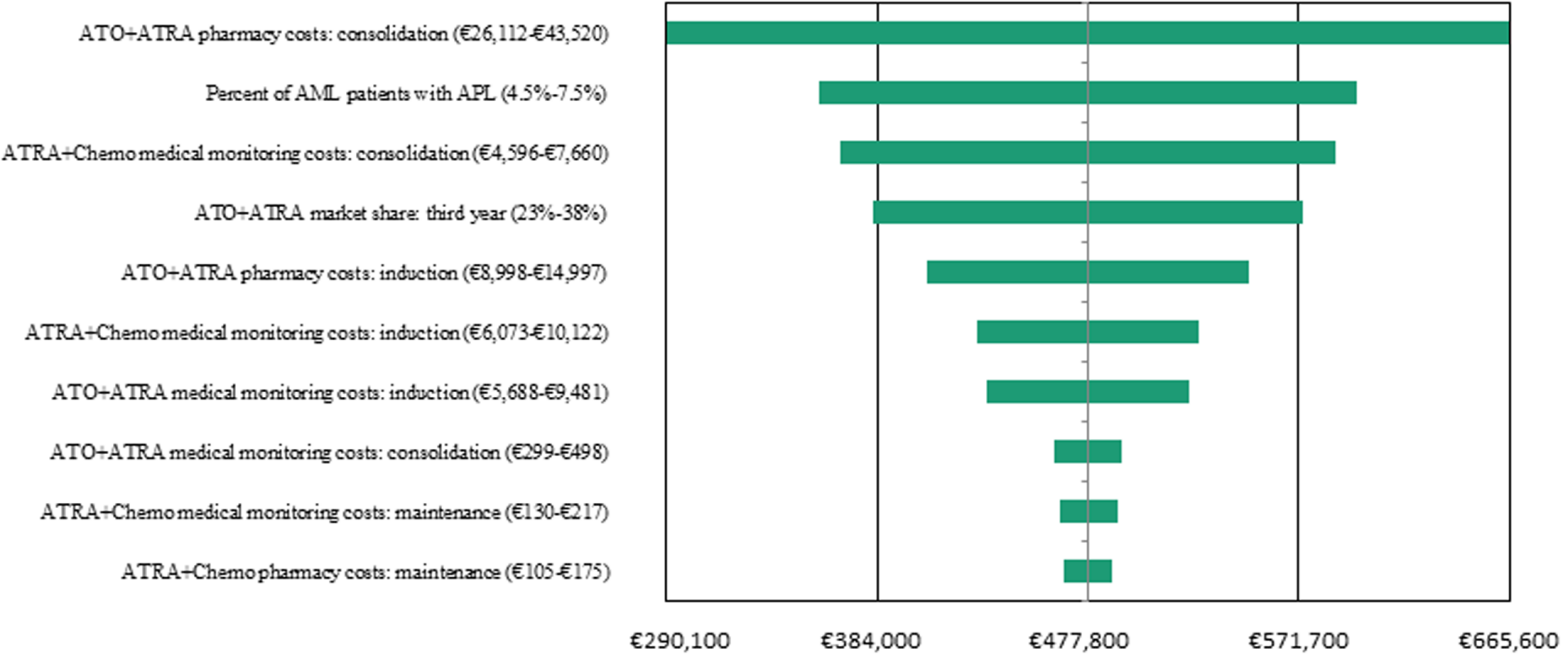
One-way sensitivity analyses based on the third-year budgetary impact. The estimated model was most sensitive to changes in the cost of ATO in the consolidation phase, in parameters determining the number of patients with APL, and in the cost of in-patient treatment and monitoring of AIDA patients during consolidation.

The results of the one-way sensitivity analyses are shown in Fig 3 as a tornado diagram, which displays the 10 most sensitive parameters based on the third-year budgetary impact. The model was most sensitive to changes in the cost of the ATO+ATRA regimen during the consolidation phase, in parameters determining the incidence of APL, and in the cost of in-patient treatment and monitoring of ATRA+chemotherapy patients during consolidation. When the consolidation cost of the ATO+ATRA regimen was varied between €26,112 and €43,520 (+/-25% of the base case value), the associated third-year budgetary impact ranged from €290,100 to €665,600.

## Discussion

Budgetary impact models are valuable to healthcare payers as they make formulary decisions related to the access and affordability of new health technology. Such assessments involve comparing estimated healthcare costs before and after the introduction of the intervention. In the analysis described here, the current scenario in which ATO is not available as a first-line option for patients with APL is compared with a future scenario in which ATO is available for this line of treatment. The total budgetary impact of ATO added on to ATRA was estimated in terms of its net cost, reflecting both the potential increase in drug costs and decrease in medical costs (from improved treatment outcomes, such as reductions in disease event or death) associated with its use.

We constructed a Markov state-transition model from the perspective of the overall Italian healthcare system. APL has its highest incidence in southern Europe and Latin America [2] and the clinical trial we derived much of the data from was comprised approximately 75% of Italian patients [19]. Thus, it was most relevant and consistent with real-world economics to use Italy as a base country for this analysis. The cost of treatment analysis showed that while the ATO+ATRA regimen pharmacy costs were higher than the ATRA+chemotherapy regimen pharmacy costs, all other cost components (medical, AE and disease event) were lower for ATO+ATRA patients. This meant that even though the cumulative 3-year pharmacy costs for patients treated with the ATO+ATRA regimen were approximately 7 times higher than for patients treated with the ATRA+chemotherapy regimen, the total costs associated with ATO+ATRA were only 1.5 times greater by the end of the model’s 3-year time horizon. Non-pharmacy medical costs were lower in ATO+ATRA patients for both the induction and consolidation phases. The non-pharmacy cost savings stemmed primarily from the requirement that the consolidation phase for patients receiving ATRA plus chemotherapy has to take place in a hospital setting compared to outpatient consolidation for patients on the ATO+ATRA regimen. ATO+ATRA patients have no maintenance costs (pharmacy or medical). Additionally, ATO+ATRA regimen patients have lower clinical AE risks and a shorter treatment duration (8 months versus 28 months for ATRA+chemotherapy), which could lead to non-pharmacy cost savings. This in turn also has the potential to improve patients’ quality of life, an important consideration in the treatment of long-term illness that is outside the scope of formal budgetary impact analysis. Patients receiving ATO+ATRA have reported significantly less fatigue at the end of induction than patients treated with ATRA+chemotherapy [34]. Finally, the cost of treatment analysis reported that for both regimens, the majority of the per-patient costs occur in the first year. This is due to the significant health resource utilization associated with first-line APL treatment (including pharmacy and medical) during induction therapy where the objective is to obtain complete hematologic remission, as well as during several cycles of consolidation therapy with the goal of achieving molecular complete remission.

The average budgetary impact over 3 years was €305,900 per year. To our knowledge, there have not been any published reports of the budgetary impact of the treatment of leukemia in Italy to which we could compare the results of our analysis. However, one study looked at the budget impact of the third-generation aromatase inhibitors anastrozole and letrozole as alternatives to tamoxifen in postmenopausal women with advanced breast cancer from the perspective of the Italian national health system [35]. Both anastrozole and letrozole were found to be cost-effective compared to tamoxifen for first-line therapy and were estimated to increase the budget for advanced breast cancer care by 12% and 18%, respectively.

It was not surprising that the model was most sensitive to changes in the cost of the ATO+ATRA regimen during the consolidation phase. By varying the cost by 25% of the base case, the third-year net budget impact ranged from €290,100 to €665,600. The pharmacy costs associated with first-line ATO added to ATRA, combined with the efficacy of this treatment and the need for up to 4 consolidation cycles (28 weeks), represent the largest portion of expenditures. Changes in the cost of the ATO+ATRA regimen during the induction phase represented the model’s fifth most sensitive parameter, mostly due to the shorter duration of induction as compared to consolidation. Varying the inputs underlying the incidence of APL has the potential to substantially change the size of the eligible population and thus the net budget impact. The remaining parameters that the model was most sensitive to were related to the cost of more prolonged in-patient treatment (for both induction and consolidation) and monitoring (which included maintenance) in the ATRA+chemotherapy setting, as well as ATO+ATRA product utilization / market share during year 3.

### Limitations

This analysis has several limitations related to the input data used to populate the model. Due to a lack of published data, estimates of resource use during regular clinical treatment of APL were based on clinical expert opinion and could reasonably be expected to vary, with an associated impact on costs. The clinical parameters were taken from a single phase 3 randomized clinical trial conducted in the EU; the extent to which these values will translate to a real-world Italian setting is unknown. Second, the cost of possible supportive measures to manage potential complications [2] was not included in the present analysis. Third, this analysis only included the estimated 71% of APL patients with low to intermediate risk disease. Previous research has reported poorer outcomes with ATO regimens in patients with high-risk disease [20,36]. Fourth, the current analysis is based on a 3-year time horizon since the purpose of a budgetary impact model is to aid payers in their short-term budget forecasting; therefore any costs falling outside of this time horizon are not included. Finally, as ATO is not currently approved in Italy for the first-line treatment of newly diagnosed APL patients, it is not yet clear what a specific treatment regimen that includes first-line ATO for APL may look like.

### Conclusions

This paper summarizes the methods and results of a budgetary impact model of the introduction of ATO for first-line treatment of APL patients in the Italian setting. These models are valuable to payers making formulary decisions regarding the access and affordability of new medicines. The cost of treatment analysis showed that pharmacy costs for ATO+ATRA were higher than for ATRA+chemotherapy, while all other evaluated costs were lower for ATO+ATRA treated patients. The average budgetary impact was estimated to be €305,900 per year overall, representing a 3.5% increase. These budgetary impact results were sensitive to changes in several input parameter values, most important was the cost of the ATO+ATRA regimen during the consolidation phase of treatment. Further research is needed to determine the cost-effectiveness of first-line ATO added to ATRA compared to the current standard of care, especially in light of new studies supporting better clinical and quality of life outcomes for APL patients when the treatment regimen does not contain cytotoxic chemotherapy [19,34].

## Supporting Information

S1 File BIM—Italy Data.xls. (XLS)
